# Clinical research data sharing: what an open science world means for researchers involved in evidence synthesis

**DOI:** 10.1186/s13643-016-0334-1

**Published:** 2016-09-20

**Authors:** Joseph S. Ross

**Affiliations:** 1 Section of General Internal Medicine and the Robert Wood Johnson Foundation Clinical Scholars Program, Yale School of Medicine, New Haven, CT USA; 2 Department of Health Policy and Management, Yale School of Public Health, New Haven, CT USA; 3 Center for Outcomes Research and Evaluation, Yale–New Haven Hospital, New Haven, CT USA

**Keywords:** Data sharing, Systematic reviews, Clinical trials

## Abstract

The International Committee of Medical Journal Editors (ICMJE) recently announced a bold step forward to require data generated by interventional clinical trials that are published in its member journals to be responsibly shared with external investigators. The movement toward a clinical research culture that supports data sharing has important implications for the design, conduct, and reporting of systematic reviews and meta-analyses. While data sharing is likely to enhance the science of evidence synthesis, facilitating the identification and inclusion of all relevant research, it will also pose key challenges, such as requiring broader search strategies and more thorough scrutiny of identified research. Furthermore, the adoption of data sharing initiatives by the clinical research community should challenge the community of researchers involved in evidence synthesis to follow suit, including the widespread adoption of systematic review registration, results reporting, and data sharing, to promote transparency and enhance the integrity of the research process.

## Background

In January 2016, the International Committee of Medical Journal Editors (ICMJE) announced a bold proposal to require data generated by interventional clinical trials that are published in its member journals to be responsibly shared with external investigators, both to fulfill an ethical obligation to the research participants and to enhance the integrity of the clinical research process by enabling independent confirmation of results [1]. In response, more than 300 comments were submitted by researchers and organizations, at least some of which were unsupportive [2]. Thus, while any guidance from the ICMJE on this issue is yet to be finalized, we can reflect upon what the overall movement toward a research culture that supports data sharing might mean for the community of researchers involved in evidence synthesis and the design, conduct, and reporting of systematic reviews and meta-analyses.

## Main text

Support for data sharing as a means for improving the reliability of medical evidence and reinforcing evidence-based practice has grown steadily over the past 5 to 10 years. Over this time, several major research funders, including the US National Institutes of Health, the US Patient-Centered Outcomes Research Institute, the UK Medical Research Council, and the Bill and Melinda Gates Foundation, have adopted policies supporting or mandating clinical research data sharing [3, 4]. Private industry, by far the biggest funder of clinical research, has similarly adopted supportive policies [5], perhaps even stronger than those adopted by government and non-profit funders [6]. Academic clinical trialists, while expressing some concerns, have also demonstrated growing support for data sharing [7–9].

In January 2015, the Institute of Medicine (IOM) of the US National Academies further strengthened these efforts with its report, “Sharing Clinical Trial Data: Maximizing Benefits, Minimizing Risks,” recommending that stakeholders foster a culture in which data sharing is the expected norm and commit to responsible strategies aimed at maximizing benefits, minimizing risks, and overcoming challenges of sharing clinical trial data [10]. The 2016 ICMJE proposal clearly aligns with the IOM recommendations, requiring as a condition of consideration for publication of a clinical trial report in a member journal that the authors share with others the de-identified individual patient-level data (IPD) underlying the results presented in the article within 6 months of publication and include a plan for data sharing as a component of clinical trial registration.

A clinical research infrastructure that requires data sharing, and is increasingly transparent, holds great promise for the future of evidence synthesis [11]. Among the many challenges to rigorous systematic review and meta-analysis is non-publication of research, prohibiting the identification and inclusion of all relevant research. In addition to selective publication, other challenges include selective outcome reporting and lack of published data for potentially relevant patient subgroups. As prior studies have demonstrated, somewhere on the order of one half to one third of clinical research studies are never published [12–18], and far fewer report all collected endpoints of clinical importance, especially safety endpoints [19–21]. Data sharing may help to address publication and outcome reporting biases to improve the accuracy of systematic reviews and ensure that each is based on all relevant research studies. Researchers involved in evidence synthesis will now be able to request access to the IPD from a clinical trial to investigate briefly reported safety endpoints or examine efficacy endpoints among important subgroups, allowing fuller examination treatment effect heterogeneity for patient populations of potential interest to clinical communities, professional organizations, and guideline committees. However, it is important to note that the ICMJE proposal only applies to clinical trials published in member journals; for unpublished clinical research to be identified and included in systematic reviews, researchers will continue to rely upon research funders’ data sharing initiatives as well as trial registration and results reporting requirements.

At the same time, a clinical research infrastructure that requires data sharing poses challenges for evidence synthesis. First, the universe of applicable databases that need to be searched to identify all relevant research continues to expand, potentially taxing researchers or requiring the involvement of information specialists as they scrutinize a multitude of sources that now includes data sharing platforms, such as DRYAD, ClinicalStudyDataRequest.com, and the Yale University Open Data Access Project [22–24]. This widening universe also requires that investigators carefully cross-check among identified trials, to ensure that the same trial is not erroneously included multiple times because of discordant information reporting in multiple databases. Second, for published trials, the ICMJE proposal clearly states that all de-identified IPD underlying the results presented in the article are required to be shared. However, the availability of IPD might be considered a double-edged sword, as researchers may now face the burden of validating published trial results using IPD prior to including the summary findings in a systematic review or meta-analysis. Third, the proposal is silent on IPD from the clinical trial that does not underlie results presented in the article. For instance, this requirement could be interpreted to preclude sharing of data on the efficacy and safety outcomes, whether common or rare, not reported in the main trial publication, as well as results for patient subgroups that were not reported. Researchers must still do the legwork to obtain information on these unpublished outcomes and patient subgroups. Fourth, the ICMJE proposal may lead to a situation in which authors who publish multiple articles from a trial prepare multiple IPD files from the same clinical trial, creating version control issues and potential confusion among researchers who attempt to reconcile trial findings across the multiple IPD files. Related to this point, the availability of results from multiple sources, including articles, trial registries, and IPD, necessitates that researchers involved in evidence synthesis resolve discrepancies that are likely to exist among all reported results from included individual trials [25, 26].

A larger challenge also exists for the evidence synthesis community, including organizations such as the Cochrane Collaboration that have long advocated for stronger data sharing policies [27]. These researchers should now take on the same challenges being imposed on clinical trial researchers, enhancing efforts in the field to promote transparency and share clinical research data. As a condition of publication, in 2005, the ICMJE began requiring clinical researchers to prospectively register clinical trials in a public trial registry that allowed reporting of key trial information and pre-specification of primary and secondary outcomes, as well as safety endpoints [28]. While adherence to this policy has not been perfect [29–31], it led to remarkable increases in clinical trial registration [32] and made clinical research far more transparent. A similar effort to require systematic review registration was begun in 2012 in order to reduce unplanned duplication of reviews, provide transparency, and minimize reporting bias [33]. Efforts to ensure adherence to these requirements are needed, as the ICMJE does not currently require prospective systematic review registration as a condition of publication and some issues in primary outcome reporting have been identified [34]. However, with more than 10,000 registered systematic reviews in the largest international public registry [35], the field is clearly moving toward a more transparent scientific process.

Evidence synthesis researchers and organizations should similarly adopt policies supporting results reporting and research data sharing. The ICMJE has supported trial results reporting since 2007, when the US Food and Drug Administration Amendments Act initiated the requirement for medical product intervention trials, clarifying that brief, structured results posted in the same clinical trials register in which the initial registration resides would not be considered a prior publication [36]. Systematic review registries should be modified to accommodate results reporting, not only linkages to published articles, so that the results of unpublished systematic reviews can be better disseminated throughout the research community. Time frames for results reporting should also be specified, ideally within 12 months of systematic review completion or to coincide with publication, whichever comes first. A structured format for the reporting of results should be developed by researchers and stakeholder organizations in the field.

Efforts should also be undertaken to facilitate the sharing ﻿of libraries of articles that were aggregated for systematic reviews and meta-analyses, along with the results, summary data, and IPD that underlie the work. Resources will need to be allocated to create repositories for this information, such as the Open Science Framework [37], which could also potentially be managed by systematic review registries. To overcome copyright issues, aggregated articles might be listed on registries with linkages to the publications at journal publishers’ websites or PubMed at the US National Library of Medicine. Such a system is quite similar to how institutional repositories currently work and, if linked with publishers’ websites, might encourage publishers to enhance publications’ meta-data and facilitate retrievability and usability. To overcome IPD data ownership issues, explicit details from where and how data access was obtained could be provided, including linkages to data sharing repositories. Time frames for sharing aggregated articles, results, summary data, and IPD sharing should be specified and align with those recommended by the IOM and proposed by the ICMJE, and formats for distribution will need to be standardized. Finally, as in the clinical research community, some means of providing appropriate credit to those researchers sharing data needs to be developed and recognized in the academic community. While each of these suggestions to share data requires additional time and effort on the behalf of researchers, these are the same burdens that have been imposed on clinical trial researchers. Moreover, one could argue that the potential to reduce unplanned duplicative searching, aggregation, and analysis in the evidence synthesis community is likely far greater than the potential to reduce unplanned duplicative clinical trials in the clinical research community, although the burden of the latter is greater because it involves patients.

## Conclusion

Data sharing is about more than minimizing duplicative data collection efforts. Sharing reduces research costs and lowers human participant burden. Sharing maximizes the value of collected data by enabling follow-up studies of secondary research questions by a multitude of investigators using existing data. Sharing encourages multiple examinations and interpretations of data, both protecting against faulty analyses and contributing to replication, refinement, or refutation of prior work. Sharing positions research data as a public good [38]. The entire biomedical research community, from the basic sciences to the clinical sciences to the evidence synthesis sciences, has much to be gained from data sharing efforts. The ICMJE proposal is the next important step in the overall movement toward a research culture that supports data sharing. The community of researchers involved in evidence synthesis should embrace the challenges being imposed on clinical trial researchers in order to promote transparency and share research data.

## Abbreviations

ICMJE: International Committee of Medical Journal Editors
IOM: Institute of Medicine
IPD: Individual patient-level data
